# Neuromodulation in Neuro-Oncology: A Scoping Review

**DOI:** 10.3390/jpm16070349

**Published:** 2026-06-28

**Authors:** Ahmad I. Kamaludin, Ashwin Kumaria, Keyoumars Ashkan

**Affiliations:** 1 Departments of Neurosurgery, King’s College Hospital, London SE5 9RS, UK; 2 National Hospital for Neurology and Neurosurgery, London WC1N 3BG, UK

**Keywords:** neuromodulation, neuro-oncology, brain tumour, cancer neuroscience, glioma

## Abstract

**Background:** Neuromodulation is a rapidly developing field with growing interest in its application in neuro-oncology, particularly since the publication of the EF-14 trial which demonstrated a survival benefit conferred by tumour treating fields (TTF) in patients with glioblastoma. In addition, the emerging field of cancer neuroscience has postulated the role of neural–tumour communication in tumour aetiology, which is theoretically targetable by neuromodulation strategies. This scoping review therefore aims to comprehensively evaluate current or future applications of neuromodulation in managing patients with brain tumours, encompassing preclinical and clinical studies. **Methods**: The MEDLINE database was queried for all relevant articles from inception to 1 December 2024. A synthesis of findings was performed, broadly categorised to preclinical and clinical research. **Findings:** The database search returned 3296 results, from which 187 full-text articles were further assessed. A total of 79 studies met the inclusion and exclusion criteria and were included. The results from preclinical studies (*n* = 18) were stratified according to modality which included electrical therapy, electroporation, electromagnetic field (EMF) and deep brain stimulation (DBS). Similarly, clinical studies (*n* = 61) were classified to preoperative modalities such as transcranial magnetic stimulation (TMS) and transcranial direct stimulation (tDCS), and postoperative modalities such as TMS, TTF, EMF and spinal cord stimulation (SCS). **Interpretation:** The application of neuromodulation as adjunctive therapy in the context of neuro-oncology is an emerging field, with encouraging results in various modalities across a wide range of applications from surgical planning and functional rehabilitation, to its therapeutic potential. Further research is urgently needed to harness the potential of neuromodulation in improving patient outcomes.

## 1. Introduction

Brain and central nervous system tumours remain a major cause of morbidity and mortality [1]. Glioblastoma (GBM), the most aggressive primary brain cancer, has a poor prognosis with inevitable recurrence [2]. Exploring novel treatments, from personalised immunotherapy to neuromodulation, is crucial [3,4]. Neuromodulation, a rapidly evolving field, uses implantable or non-invasive technologies to modulate neural activity via electrical, electromagnetic, or chemical methods [5]. Its role in neuro-oncology grew after the EF-14 trial, which showed improved survival in GBM patients receiving electrical field therapy with temozolomide [6,7].

Research into neuromodulation for brain tumours suggests mechanisms including mitotic suppression, apoptosis upregulation, and increased reactive oxygen species toxicity [8,9,10]. Cancer neuroscience theories highlight neural–tumour interactions—via glutamatergic transmission, neuroligin-3, and neurotransmitter receptors—suggesting a role for neuromodulation in tumorigenesis [11,12,13,14]. Early-stage studies on other modalities such as electromagnetic fields and SCS have reported a promising outlook [15,16]. Beyond treatment, neuromodulation also aids in surgical planning. Transcranial magnetic stimulation (TMS) enhances preoperative mapping of the eloquent cortex and subcortical pathways [17], while preoperative electrical stimulation induces cortical plasticity and functional reorganisation, aiding eloquent cortex resection [18,19]. Similar techniques support recovery after surgery, stroke, and head trauma [20,21]. Emerging evidence links neuronal activity to non-CNS tumour growth, expanding neuromodulation’s potential applications [22,23]. Indeed, there may be other types of brain tumours that can also benefit from neuromodulation.

This scoping review assesses the literature on neuromodulation in neuro-oncology as an adjunct to existing therapies, covering both preclinical and clinical settings, to evaluate its current and future roles in managing brain tumours.

## 2. Methods

This scoping review was conducted in accordance with the PRISMA Extension for Scoping Reviews (PRISMA-ScR) guidelines (Supplementary File S1).

### 2.1. Protocol and Registration

A review protocol was developed prior to study commencement to guide the objectives, eligibility criteria, search strategy, and methodological approach. The protocol was not prospectively registered. As this study was conducted as a scoping review, formal registration was not mandatory; however, a predefined methodological framework was established before study initiation to enhance methodological rigour, transparency, and consistency throughout the review process. The absence of prospective registration is acknowledged as a limitation, as it may reduce external verification of protocol adherence and introduce the potential for reporting bias.

### 2.2. Eligibility Criteria

This review examined the application of neuromodulation in neuro-oncology. For the purposes of this review, neuromodulation was defined as any intervention that directly or indirectly modifies central nervous system (CNS) activity through electrical or electromagnetic stimulation.

The review adopted a broad conceptual definition of neuromodulation to include both invasive and non-invasive modalities capable of altering neuronal excitability, tumour microenvironment signalling, or tumour cell proliferation through bioelectrical mechanisms.

Eligible interventions included, but were not limited to:

- Deep brain stimulation (DBS).
- Vagus nerve stimulation (VNS).
- Spinal cord stimulation (SCS).
- Occipital nerve stimulation.
- Transcranial magnetic stimulation (TMS).
- Transcranial direct current stimulation (tDCS).
- Tumour treating fields (TTF).
- Pulsed radiofrequency therapies.
- Other electrical or electromagnetic field-based interventions applied in a neuro-oncological context, including peripheral nerve stimulation.

Studies were included if they:

- Investigated primary or metastatic brain tumours.
- Included adult or paediatric populations.
- Used in vitro or in vivo experimental models.
- Examined therapeutic, mechanistic, or safety outcomes.

Eligible study designs included:

- Randomised controlled trials.
- Non-randomised interventional studies.
- Cohort studies.
- Case series.
- Preclinical Experimental Research.

Exclusion criteria:

- Editorials, commentaries, review articles, and case reports.
- Studies focused solely on device engineering or technical development without biological or clinical outcomes.
- Non-English publications.

### 2.3. Information Sources and Search Strategy

A systematic search was conducted to identify studies examining neuromodulation in neuro-oncology. MEDLINE (via Ovid) was searched from database inception to 1 December 2024, with no date restrictions applied. To ensure comprehensive coverage, additional searches were performed in EMBASE, Web of Science, Scopus, and Cochrane CENTRAL. Clinical trial registries, including ClinicalTrials.gov and the WHO International Clinical Trials Registry Platform (ICTRP), were also consulted to capture ongoing or unpublished studies.

The search strategy combined controlled vocabulary (MeSH for MEDLINE, EMTREE for EMBASE) and free-text terms related to neuromodulation modalities (e.g., TMS, tDCS, DBS, TTF, EMF, SCS) and brain neoplasms (primary and metastatic). Terms were iteratively refined to maximise sensitivity while minimising irrelevant results. Reference lists of included studies were hand-searched to identify additional eligible articles.

All records were imported into Rayyan QCRI for screening, with duplicates removed prior to title/abstract and full-text review [24]. Two reviewers independently screened studies against predefined eligibility criteria, and disagreements were resolved through discussion or consensus. A PRISMA flow diagram was generated to illustrate study selection.

#### Search Strategy

exp Electric Stimulation Therapy/or Electric Stimulation Therapy.mp. or (exp Deep Brain Stimulation/or Deep Brain Stimulation.mp.) or (exp Electric Countershock/or Electric Countershock.mp.) or (exp Electroacupuncture/or Electroacupuncture.mp.) or (exp Pulsed Radiofrequency Treatment/or Pulsed Radiofrequency Treatment.mp.) or (exp Spinal Cord Stimulation/or Spinal Cord Stimulation.mp.) or (exp Transcranial Direct Current Stimulation/or Transcranial Direct Current Stimulation.mp.) or (exp Transcutaneous Electric Nerve Stimulation/or Transcutaneous Electric Nerve Stimulation.mp.) or (exp Vagus Nerve Stimulation/or Vagus Nerve Stimulation.mp.) or tum*r treating fiel*.mp. or (exp Transcranial Magnetic Stimulation/or Transcranial Magnetic Stimulation.mp.) or (exp Electromagnetic Fields/or Electromagnetic Fields.mp.) or (exp Electromagnetic Radiation/or Electromagnetic Radiation.mp.) or (deep brain stimulation or vag* nerve stimulation or transcranial magnetic stimulation or tum*r treating fiel* or electr* fiel* or electromagnetic radiation).mp.

And exp Brain Neoplasms/or Brain Neoplasms.mp. or (brain neoplasm or brain cancer or brain tum*r or glioma or glioblastoma or neuro-oncology or neurooncology or brain metas* or astrocytoma or medulloblastoma or pineo* or glioneuronal or neuroepithelial or gangloglioma).mp.

### 2.4. Study Selection

All records were imported into Rayyan QCRI for screening. After removal of duplicates, two reviewers (AIK and AK) independently screened titles and abstracts against predefined eligibility criteria.

Full-text articles were subsequently retrieved and independently assessed for inclusion. Discrepancies were resolved through discussion and consensus.

**Figure 1:**
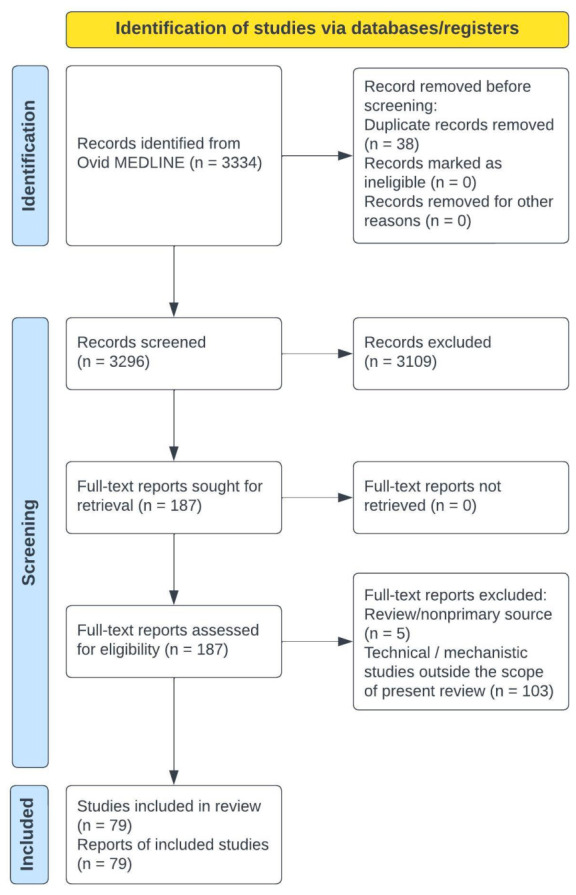
Flowchart detailing study screening and selection.

A PRISMA flow diagram was generated to illustrate the study selection process (Figure 1).

### 2.5. Data Charting Process

Data were extracted using a standardised data charting form developed by the authors. The form was piloted on a subset of included studies and refined prior to full data extraction.

Data extraction was performed by one reviewer (AIK) and verified by a second reviewer (AK) to ensure accuracy and completeness.

The following variables were considered:

- Study characteristics (author, year, country).
- Study design.
- Population characteristics (age group, tumour type).
- Intervention modality and parameters.
- Comparator (if applicable).
- Outcomes assessed (e.g., tumour progression, survival, symptom control, mechanistic endpoints).
- Safety and adverse events.
- Key findings.

### 2.6. Critical Appraisal

As this was a scoping review aimed at mapping the breadth of available evidence, a formal risk-of-bias assessment was not undertaken. However, study design and methodological limitations were considered descriptively when interpreting findings.

### 2.7. Data Synthesis

Given the anticipated heterogeneity in study design, intervention type, and outcome measures, quantitative meta-analysis was not appropriate. Findings were synthesised narratively and grouped according to neuromodulation modality and study type (preclinical vs. clinical).

### 2.8. Role of the Funding Source

The authors received no financial support for the research and authorship of this review.

## 3. Results

### 3.1. Literature Search and Study Selection

The initial database search yielded 3334 results, screened for eligibility. Of 187 full-text articles assessed, 79 were included (Figure 1). These were categorised as preclinical (18/79, 23%) and clinical studies (61/79, 77%). Among clinical studies, most focused on TMS (29/61, 48%) and TTF (25/61, 41%), with the remainder on continuous cortical electrical stimulation (*n* = 1), tDCS (*n* = 2), and EMF (*n* = 3). The characteristics and key findings of included studies are summarised in Table 1.

### 3.2. Preclinical

#### 3.2.1. Electrical Therapy

Preclinical studies on neuromodulation in GBM include electrical field therapy, electroporation, electromagnetic fields, and deep brain stimulation (DBS). Five studies focused on electrical field therapy, three of which examined Intratumoural Modulation Therapy (IMT) [25,26,27]. IMT, a novel electrotherapy using implanted low-intensity electric fields, was tested in vitro and in vivo, showing reduced tumour volume, increased apoptosis, and enhanced GBM cell death when combined with temozolomide (TMZ). Clinically, IMT could bypass scalp and cranial shunting, enabling direct electrical delivery while minimising TTF-associated skin toxicity. Wu et al. evaluated the TEFTS ASCLU-300 adjustable electrical field system, showing a 16-day survival increase (40 vs. 24 days) in glioma-transplanted rats and reduced tumour size [28]. Similarly, Bardet et al. studied nanosecond pulsed electrical fields (nsPEFs), delivering high-voltage pulses that caused vascular collapse and cell death in GBM organoid models, demonstrating therapeutic potential [29]. However, these findings remain largely limited to controlled preclinical models, which may not fully replicate the complexity and heterogeneity of human GBM. Consequently, the translational potential and clinical efficacy of these neuromodulation strategies require further validation.

#### 3.2.2. Electroporation

Electroporation, a type of electrochemotherapy, uses electrical pulses to increase blood–brain barrier permeability for drug delivery (reversible) or cause nonthermal cell membrane damage and cell death (irreversible) [30]. Reversible electroporation has been shown to increase chemotherapeutic efficacy in gliomas [30,31,32]. In rodents with GBM, Sharabi et al. reported a seven-day median survival increase (15 vs. 22 days) with electroporation and cisplatin versus cisplatin alone [31]. Agerholm-Larsen et al. found 69% of rodents treated with electroporation and bleomycin achieved complete tumour elimination, in contrast to none in controls [30]. For irreversible electroporation (IRE), Latouche et al. developed high-frequency IRE (H-FIRE), using short bipolar pulses to minimise muscle contractions and selectively ablate tumour cells [33]. In rats with implanted F98 glioma cells, H-FIRE combined with chemotherapy significantly improved survival compared to controls [34]. Taken together, electroporation may synergise the anti-cancer effects of chemotherapy.

#### 3.2.3. Electromagnetic Fields

Electromagnetic fields (EMF) show anticancer effects in vitro and in vivo [35]. Six studies examined EMF in neuro-oncology. Radiofrequency EMF increased blood–brain barrier permeability tenfold in vitro, suggesting enhanced drug delivery and treatment efficacy [36]. Combined with temozolomide (TMZ), EMF increased apoptosis by up to 50% and doubled reactive oxygen species in glioma cell lines (T98G and U87G) [37]. EMF and TMZ also induced apoptosis in chemoradiotherapy-resistant T98G cells, though with limited effect on proliferation [38]. Helekar et al. investigated a novel oscillating magnetic field device, which selectively killed GBM cells while sparing normal tissue [39]. However, EMF effects vary by frequency and amplitude—some inhibit GBM proliferation, while others stimulate it—highlighting the need for caution in clinical translation [9]. Across the studies there appears to be interest in determining optimal parameters for an anti-tumour effect.

#### 3.2.4. Deep Brain Stimulation

A preclinical study explored DBS as an antitumour treatment, leveraging its familiarity as an implanted system [40]. Using DBS electrodes similar to those in Parkinson’s disease, researchers tested various frequencies on brain tumour cell lines over seven days, observing significant metabolic reduction via G0-phase cell cycle accumulation. DBS also enhanced chemotherapy effects while sparing post-mitotic astrocytes, preserving neurologically functional cells. As with EMF, future work may define optimal parameters for a desirable anti-tumour effect while mitigating adverse effects of stimulation.

### 3.3. Clinical

#### 3.3.1. Preoperative—TMS, CCES and tDCS

Preoperatively, neuromodulation—especially TMS—supports surgical mapping, risk stratification, and outcome improvement. Over the past decade, TMS has been used to functionally map motor and language centres. Six observational studies showed that navigated TMS (nTMS), combining physiological data with MRI, localises eloquent regions, refines resection plans, clarifies anatomy, and rules out suspected motor or language cortex involvement [41,42,43,44]. While nTMS maps only cortical areas, combining it with diffusion tensor imaging fibre tracking (DTI-FT) allows visualisation of functional white matter tracts, useful when awake surgery with intraoperative neurophysiological monitoring (IONM) is not feasible [45,46]. nTMS mapping reconstructed the language cortico-subcortical network and aided neuronavigation, mainly in adults. In children under six, TMS was effective and well-tolerated, though 27% of epileptic patients had peri-TMS seizures, none requiring intervention [47].

Ten studies compared preoperative TMS mapping versus no mapping—eight on motor-eloquent and two on language regions. TMS patients had higher gross total resection rates (59–92.3% vs. 42–78.4%), though two studies found no significant difference [48,49,50,51,52,53,54]. Overall survival (OS) varied—one study showed significantly longer survival (22.4 vs. 15.4 months), while another found non-significant improvement (15.7 vs. 11.9 months) [48,53]. In subgroup analysis, WHO Grade 3 glioma patients had significantly better OS (21.5 vs. 7.2 months), unlike Grade 4 cases [53]. Hendrix et al. alternatively reported no difference in the survival rates of malignant glioma patients between nTMS and non-nTMS groups [49]. Motor outcomes were better in nTMS-mapped motor-eloquent lesions, with higher improvement rates (12–30.8% vs. 1–13.1%), better postoperative MRC scores, and reduced deterioration (3.4% vs. 13.1%). Other studies reported similar rates of permanent paresis and motor recovery. For language-eloquent lesions, long-term outcomes were similar between rTMS and non-rTMS groups, though rTMS patients had better immediate postoperative results that equalised over time [50,55].

Interest is growing in TMS for risk stratification and prognosis of postoperative function. Traditionally, surgical risk is based on tumour location, with eloquent areas carrying the highest morbidity [56]. However, emerging evidence suggests anatomical location alone is insufficient for risk assessment and ascertaining eligibility for surgery [57]. Four studies examined nTMS tractography as a predictor of postoperative motor function. Resection of TMS-positive points significantly predicted permanent deficits, while precentral gyrus resection did not [58,59]. nTMS tractography showed higher predictive value than anatomic tractography. Greater distance between tumours and abductor pollicis brevis hotspots, mapped via nTMS and motor-evoked potentials, correlated with better grip strength recovery at three months [60]. Similarly, no permanent deficits were recorded when tumour–corticospinal tract (CST) distance exceeded 8–12 mm, as identified by nTMS and fibre tracking [61,62]. One study assessed nTMS mapping for arithmetic processing (AP) centres, linking AP-positive site resection with postoperative arithmetic decline [17]. However, false positives occurred: 4/8 patients had TMS-positive sites resected without deficits, and 3/15 had AP-positive sites removed with paradoxical improvement.

Functional reorganisation from tumour-induced plasticity is a novel area, first noted in intraoperative language mapping during repeat awake craniotomies [63,64]. Preoperative detection of reorganisation may influence surgical timing, especially in low-grade tumours, where surgery could be delayed to allow cortical and subcortical function to reorganise [65]. nTMS demonstrated significant cortical motor shifts in most patients when measured longitudinally, pre- and postoperatively [63]. Another study found increased language-negative sites in tumour areas on postoperative nTMS, suggesting functional migration away from the tumour [66]. Beyond detection, neuromodulation may facilitate functional reorganisation. Rivera et al. applied a prehabilitation protocol using an implanted subdural grid with daily cortical electrical stimulation (CCES) and intensive behavioural training [18]. In five patients with eloquent tumours, this strategy induced intra-tumoral functional displacement, allowing more extensive resections, confirmed by fMRI. Similarly, a pilot study of tDCS with right-handed motor training in eight glioma patients increased left primary motor cortex connectivity, with no adverse events [19]. These findings highlight the potential of electrical stimulation in modulating neural networks. As modulation of neural networks is one of the key concepts in cancer neuroscience [11,12,13,14], one can be optimistic as further reports are awaited.

#### 3.3.2. Postoperative—TMS

Postoperatively, neuromodulation ranges from emerging approaches like TMS for neurorehabilitation and SCS to modify the tumour microenvironment, to established treatments such as TTF. In stroke, rTMS aids neurorehabilitation through cortical reorganisation with minimal seizure risk [90]. Research on TMS in neuro-oncology is nascent, with three studies involving 60 patients. A small randomised study (n = 22) examined seven days of low-frequency navigated rTMS (nrTMS) versus sham stimulation [21]. The primary outcome, change in the Fugl–Meyer Assessment score, showed a significant increase (31.9 vs. 4.2), surpassing the clinically significant threshold (>10). A smaller study (*n* = 7) using the same protocol found six patients regained preoperative function, with one improving beyond baseline [67]. A proof-of-concept study used a network-specific rTMS protocol, comparing lesions to connectomes (Human Connectome Project) to create targeted cortical networks for rehabilitation [68]. Treatment promoted network reorganisation, yielding clinically significant benefits in 90.3% of patients, measured by motor improvement or ≥3-point Aphasia Rapid Test reduction. Across all studies, nrTMS was safe, with no serious complications reported, although further studies are indicated to evaluate efficacy.

#### 3.3.3. Postoperative—TTF

TTF has been widely studied in two phase 3 RCTs and multiple real-world studies on outcomes, quality of life, and cost-effectiveness [6,69]. The EF-11 trial (*n* = 237), the first RCT on TTF, compared TTF monotherapy (NovoTTF-100A) with physician’s choice chemotherapy in recurrent GBM. OS was not statistically significant: 1-year survival was 20% in both groups, median OS 6.6 vs. 6 months, and 6-month PFS 21.4% vs. 15.1%. Patients completing at least one full TTF course (28 days) had improved OS (7.7 vs. 5.9 months). Two post-marketing registries, PRiDe and EF-19, followed [70,71]. PRiDe showed higher median OS with TTF (9.6 vs. 6 months), while EF-19 found benefits (8.1 vs. 6.4 months) only in patients using TTF > 18 h/day for >28 days. More PRiDe patients received TTF after first recurrence, possibly contributing to better outcomes.

This led to the EF-14 trial (*n* = 695), which randomised patients 2:1 to TTF (Optune) plus maintenance TMZ or TMZ alone [6]. PFS and OS were primary and secondary outcomes. TTF significantly improved PFS (6.7 vs. 4 months) and OS (20.9 vs. 16 months), with higher 2-year (43% vs. 31%) and 5-year survival (13% vs. 5%). In recurrent cases, post hoc analysis showed modest OS benefit with TTF plus second-line chemotherapy (11.8 vs. 9.2 months) [72]. Skin-related adverse events (36–83%) were most common, but overall TTF showed a favourable safety profile with no new concerns (post-marketing data, 11,029 patients) [73,74]. Systemic adverse events were comparable to chemotherapy alone [6]. Health-related QoL remained similar, except for scalp pruritus [75,76]. The device’s heavy battery pack may limit use, particularly in patients with balance impairment.

As with EF-11 registries, real-world analysis of TTF is important. Despite promising EF-14 results, outcomes remain heterogeneous, though two studies confirmed improved PFS and OS with TTF plus TMZ [77,78]. But others found no meaningful benefit on OS or survival rate [79,80]. Liu et al. suggested that larger patient cohorts might be necessary to detect any effect if it does exist [80]. This brings into consideration the cost-effectiveness of TTF and whether achieving a clinically significant result is economically feasible [81]. An American study estimated TTF + TMZ to cost an incremental cost-effectiveness ratio of $150,452 per life year gained, which is within the US willingness-to-pay threshold [82]. A French study criticised the analysis, reporting a much higher cost (€510,273 per life year) exceeding their willingness-to-pay threshold, and suggested TTF would need an 85% price reduction to be viable [83]. Further research is needed to optimise TTF’s efficacy and applicability. The ongoing TRIDENT (EF-32) trial is evaluating its use with chemoradiotherapy in newly diagnosed GBM, results of which are awaited [84]. Future trials exploring TTF with personalised immunotherapy will also be of interest, harnessing the potential of multimodal treatment for GBM. However, the costs of TTF remain prohibitive for the vast majority of people with brain tumours.

#### 3.3.4. Postoperative—SCS and EMF

High-grade gliomas (HGG) are characterised by high hypoxia levels, contributing to resistance to chemotherapy and radiotherapy [91,92]. Cervical SCS may modify the tumour microenvironment by increasing blood flow and reducing hypoxia, potentially enhancing adjuvant treatment efficacy [85]. A small case series (*n* = 7) investigated SCS during brain reirradiation and chemotherapy in recurrent HGG, reporting a median OS of 39 months and PFS of 19 months [16]. Despite the small sample size and single-arm study design, SCS was associated with longer survival when compared to similar reirradiation studies [16,86,87]. Another emerging modality is the Voyager System, a wearable device using magnetic fields instead of electrical stimulation, avoiding TTF’s battery weight and skin irritation [15,88,89]. Early feasibility studies show a favourable safety profile, supporting further trials to assess efficacy. Meanwhile, less invasive spinal stimulation and/or SCS at locations other than the cervical spinal cord may also be studied in the future.

## 4. Discussion

This review provides a comprehensive overview of neuromodulation across all stages of neuro-oncology, spanning preclinical and clinical studies. Unlike prior reviews, it integrates multiple modalities—including TTF, TMS, tDCS, EMF, DBS, SCS, and intratumoural electrical therapies—within a cancer neuroscience framework, linking neuronal activity, synaptic integration, and neuro-immune crosstalk to potential anti-tumour mechanisms. While earlier work focused on specific modalities or clinical applications, this review synthesises mechanistic and translational evidence and is the first to explicitly highlight neuromodulation’s potential anti-tumour effects.

Framing neuromodulation within cancer neuroscience highlights potential mechanisms linking neuronal activity to tumour progression. TTF and intratumoural electrical therapies may disrupt synaptic signalling and activity-dependent glioma growth, enhancing chemosensitivity, potentially via modulation of extracellular matrix proteins such as TSP-1 [11,12,13,14,81]. EMF could modulate calcium signalling and ROS production, indirectly affecting activity-driven proliferation [9,35,36,37,38,39]. DBS, VNS and SCS may alter network excitability and autonomic tone including modulation of adrenergic and/or parasympathetic/vagal activity [22,40]. DBS and SCS may also influence the tumour microenvironment through perfusion, hypoxia, or neuro-immune crosstalk [22,40,81]. TMS and tDCS may induce cortical plasticity and functional reorganisation, potentially modifying neuronal inputs to tumour cells and limiting proliferation or migration [18,20,66]. Considering these mechanisms provides a framework to guide translational research and rational clinical trial design.

Preclinical research shows promising results for clinical translation. Electrical therapy, magnetic fields, electroporation, and DBS inhibit brain tumour cells and may synergize with chemotherapy. However, much of this evidence derives from in vitro systems or small animal models that may not fully capture the biological heterogeneity, immune interactions, and treatment resistance observed in human gliomas. As such, the translational validity of these findings remains uncertain, and therapeutic effects demonstrated under controlled laboratory conditions may not be reproducible in clinical settings. Notably, only magnetic field therapy (Oncomagnetic) has been trialled in humans, showing good tolerance in a single recurrent GBM patient and reduced tumour volume [93]. Clinically, TMS aids preoperative planning, risk assessment, and functional recovery through cortical reorganisation. Outcomes for OS, GTR, paresis, and language are variable, though results are generally positive for neurorehabilitation [48,49,50,52]. Technical comparisons of TMS with fMRI or DCS were beyond this review’s scope. TTF remains the most researched and established neuromodulation therapy, but EF-14 trial findings did not consistently improve survival in real-world practice [79,80]. However, the interpretation of EF-14 remains controversial owing to its open-label design, lack of sham control, and concerns regarding potential sponsor-related bias. Poor cost-effectiveness, particularly in socialised healthcare systems, and provider scepticism act as barriers to widespread adoption [94].

An important consideration when interpreting the TTF literature is that, despite the positive findings of the EF-14 trial, the magnitude and generalisability of its reported survival benefit remain subjects of ongoing debate [95]. While EF-14 demonstrated significant improvements in progression-free and overall survival with the addition of TTF to maintenance temozolomide, several limitations have been highlighted, including the open-label study design, potential performance and compliance biases, the absence of a sham-control arm, and concerns regarding sponsor involvement in trial conduct and analysis [95]. These factors warrant caution when interpreting EF-14 as definitive evidence of efficacy and emphasise the importance of corroborating findings through independent studies and real-world datasets. Indeed, subsequent real-world studies have produced mixed results, with some cohorts reproducing the survival advantages observed in EF-14 while others have reported more modest benefits, suggesting that patient selection, healthcare system factors, and especially treatment adherence may substantially influence outcomes [96,97]. Nevertheless, TTF remains the most extensively investigated neuromodulation modality in glioblastoma and continues to evolve through ongoing clinical development [98]. Of particular interest are combination strategies integrating TTF with immunotherapeutic approaches, including pembrolizumab-based regimens, which aim to exploit potential synergistic effects between bioelectrical modulation and anti-tumour immune responses [99,100]. The outcomes of these studies, together with longer-term real-world experience, will be critical in defining the future role of TTF within multimodal glioblastoma management.

Although there is no evidence demonstrating benefit for paediatric patients, two small case series and two case reports assessing feasibility, safety, and compliance showed no device-related toxicities and similar compliance rates to adults [101,102,103,104]. A thinner skull and less extracranial tissue may enhance efficacy in children due to lower electrical impedance [105,106]. However, infratentorial tumour location poses additional challenges for electrical field delivery. One ongoing multicentre trial (NCT03033992) is evaluating TTF feasibility and safety in paediatric HGG and ependymoma, with full results awaited. Nevertheless, the currently available paediatric evidence is extremely limited and largely restricted to feasibility studies and case reports, which precludes meaningful conclusions regarding therapeutic efficacy. Differences in tumour biology, developmental neurophysiology, and long-term safety considerations further highlight the need for dedicated paediatric trials.

Future iteration and modification of TTF as an implantable intracranial TTF system has been proposed, which could improve efficacy by increasing field strength and stimulation time and patient comfort by eliminating skin irritation, head shaving, and external battery packs [107,108,109]. Although no studies have yet to investigate this specifically for TTF, several in vivo studies as discussed above have reported on the feasibility of an implantable IMT system delivering low-intensity electrical field in animal models with favourable results. There may be interest in the theoretical potential of alternative delivery routes, including intraspinal, intracavity, and intraventricular approaches [110].

A systematic literature search found only one study on DBS and none on VNS for brain tumour treatment, despite their established clinical use. However, their therapeutic potential is recognised and discussed previously [22,111]. Abdullahi et al. proposed VNS for cancer therapy, as it reduces oxidative stress and inflammation and activates the cholinergic anti-inflammatory pathway, potentially regulating tumour proliferation, differentiation, and apoptosis [111]. VNS may also reduce cancer growth by suppressing neural activity and modifying the systemic epigenetic landscape [22,112]. Research on DBS as an anti-cancer therapy is limited, but its established intracranial use makes it promising in neuro-oncology. Despite strong theoretical rationale, the absence of experimental and clinical studies investigating DBS or VNS specifically for brain tumour control highlights a significant gap between conceptual therapeutic potential and empirical evidence. This disparity underscores the need for mechanistic studies to clarify how neuromodulation may influence tumour biology within the complex neural–tumour microenvironment. Further studies are needed to explore its therapeutic potential.

## 5. Study Limitations

This scoping review has several limitations. The reviewed studies showed significant heterogeneity in methodology and outcomes, making thematic analysis difficult. Many studies were prone to bias due to single-arm designs with no control group, reflecting that research in this field is still emerging. There was also a lack of studies on certain neuromodulation modalities, such as occipital nerve and peripheral nerve stimulation. More generally, perhaps the biggest limitation of this topic, is that the precise mechanisms by which each neuromodulation modality exerts potential anti-tumour effects remain largely speculative. While preclinical and early clinical findings suggest modulation of neuronal activity, synaptic signalling, and microenvironmental factors, definitive causal pathways have yet to be established. As above, further studies are warranted to ensure this concept of device-related neuromodulation aligns more with cancer neuroscience.

In addition, the predominance of early-phase and exploratory studies raises the possibility of publication bias, where positive findings may be preferentially reported while negative or inconclusive results remain underrepresented. Many studies also lacked standardised outcome measures and consistent follow-up durations, limiting cross-study comparability and the ability to synthesise findings quantitatively. Finally, the rapidly evolving nature of neuromodulation technologies means that current evidence may quickly become outdated as new stimulation platforms, delivery methods, and combined therapeutic strategies emerge.

Finally, although this review followed a predefined protocol and established scoping review methodology, the protocol was not prospectively registered because registration is not routinely required for scoping reviews; nevertheless, this should be acknowledged as a methodological limitation that may reduce transparency and reproducibility.

Despite these limitations, this review provides a comprehensive overview of the current research landscape and serves as a valuable summary of the evidence on device-based neuromodulation in neuro-oncology.

## 6. Conclusions

Neuromodulation represents a rapidly evolving and potentially transformative area within neuro-oncology. This scoping review demonstrates that a wide range of neuromodulatory strategies—from established approaches such as tumour treating fields and transcranial magnetic stimulation to emerging modalities including electrical field therapy, electromagnetic fields, deep brain stimulation, and spinal cord stimulation—are being explored across the spectrum of brain tumour management, from surgical planning and neurorehabilitation to direct antitumour therapy [113].

Despite encouraging signals from preclinical and early clinical studies, the current evidence base remains limited by methodological heterogeneity, small study populations, and a predominance of exploratory research. As a result, the clinical efficacy and optimal application of many neuromodulation strategies remain uncertain. Future research should prioritise mechanistic studies of neural–tumour interactions, standardisation of stimulation parameters, and well-designed prospective clinical trials to determine therapeutic benefit.

With continued advances in cancer neuroscience and bioelectrical therapeutics, neuromodulation may ultimately become an important adjunct in the multidisciplinary management of brain tumours. However, rigorous clinical investigation will be essential to translate its theoretical and experimental promise into meaningful improvements in patient outcomes.

## Figures and Tables

**Table 1:** Summary of neuromodulation studies included in the review.

Category	Modality	No. of Studies (n)	Key References	Key Outcomes
**Preclinical**	**Electrical Therapy**	5	[25,26,27,28,29]	Intratumoural modulation therapy (IMT) reduced tumour volume, increased apoptosis, and enhanced glioblastoma (GBM) cell death when combined with temozolomide. Electrical field therapy systems demonstrated improved survival in glioma rodent models and tumour size reduction. Nanosecond pulsed electrical fields caused vascular collapse and tumour cell death in GBM organoid models.
	**Electroporation**	4	[30,31,32,33,34]	Reversible electroporation increased chemotherapy delivery across the blood–brain barrier and improved survival in GBM rodent models. Irreversible electroporation (including high-frequency IRE/H-FIRE) selectively ablated tumour cells and significantly improved survival when combined with chemotherapy.
	**Electromagnetic Fields (EMF)**	6	[9,35,36,37,38,39]	EMF increased blood–brain barrier permeability and enhanced chemotherapeutic efficacy. Increased apoptosis and reactive oxygen species were observed in glioma cell lines. Oscillating magnetic field devices demonstrated selective GBM cell death while sparing normal tissue, although effects varied depending on field parameters.
	**Deep Brain Stimulation (DBS)**	1	[40]	DBS reduced tumour cell metabolism in vitro via cell-cycle arrest (G0 phase) and enhanced chemotherapeutic effects while sparing non-proliferating astrocytes.
**Clinical—Preoperative**	**TMS/CCES/tDCS**	21	[17,18,19,41,42,43,44,45,46,47,48,49,50,51,52,53,54,55,56,57,58,59,60,61,62,63,64,65,66]	Navigated TMS (nTMS) improved localisation of motor and language cortices, refined surgical planning, and increased gross total resection rates. nTMS combined with DTI-FT improved visualisation of functional white-matter tracts. Prehabilitation using cortical stimulation (CCES) induced functional reorganisation allowing larger resections. tDCS showed increased motor cortex connectivity and potential neuroplastic effects.
**Clinical—Postoperative**	**TMS (Rehabilitation)**	3	[21,67,68]	Repetitive TMS improved postoperative motor recovery and functional outcomes in glioma patients. Studies reported clinically significant improvements in Fugl–Meyer motor scores and aphasia scores with no serious adverse events.
	**Tumour Treating Fields (TTF)**	25	[6,69,70,71,72,73,74,75,76,77,78,79,80,81,82,83,84]	Two phase III RCTs and multiple real-world studies demonstrated improved progression-free and overall survival when TTF was combined with temozolomide in newly diagnosed GBM. The EF-14 trial showed increased progression-free survival (6.7 vs. 4 months) and overall survival (20.9 vs. 16 months) compared with temozolomide alone.
	**Spinal Cord Stimulation (SCS)/EMF Devices**	4	[15,16,85,86,87,88,89]	Cervical SCS may improve tumour oxygenation and enhance responsiveness to radiotherapy and chemotherapy. Small clinical studies reported prolonged survival compared with historical controls. Magnetic field–based wearable devices (e.g., Voyager system) demonstrated favourable safety profiles in early feasibility trials.

## Data Availability

No new data were created or analysed in this study.

## Supplementary Materials

The following supporting information can be downloaded at: https://www.mdpi.com/article/10.3390/jpm16070349/s1, File S1: PRISMA-ScR Checklist.
