# Supplementary material for: Neuromodulation in Neuro-Oncology: A Scoping Review
Source: J Pers Med. 2026 Jun 28;16(7):349. doi: 10.3390/jpm16070349 (PMC13413177; doi:10.3390/jpm16070349)
Supplement: Supplementary file 1 [file jpm-16-00349-s001.zip › jpm-4320689-supplementary.pdf]

PRISMA-ScR Checklist

Manuscript Title: *Neuromodulation in Neuro-Oncology: A Scoping Review*

Reporting Guideline: PRISMA Extension for Scoping Reviews (PRISMA-ScR)

Section	PRISMA-ScR Item	Checklist Item	Reported on Page/Section
Title	1	Identify the report as a scoping review.	Title page
Abstract	2	Provide a structured summary including background, objectives, eligibility criteria, sources of evidence, charting methods, results, and conclusions.	Abstract
Introduction	3	Describe the rationale for the review in the context of existing knowledge.	Introduction
Introduction	4	State the objectives and/or questions of the review.	Introduction
Methods	5	Indicate whether a review protocol exists and where it can be accessed.	Methods – Protocol and Registration
Methods	6	Specify eligibility criteria for sources of evidence.	Methods – Eligibility Criteria
Methods	7	Describe all information sources used in the search.	Methods – Information Sources and Search Strategy
Methods	8	Present the full electronic search strategy for at least one database.	Methods – Search Strategy
Methods	9	State the process for selecting sources of evidence.	Methods – Study Selection
Methods	10	Describe the data charting process and whether forms were calibrated or tested.	Methods – Data Charting Process
Methods	11	List and define all variables for which data were sought.	Methods – Data Charting Process
Methods	12	If performed, provide rationale for critical appraisal of included sources.	Methods – Critical Appraisal
Methods	13	Describe methods for handling and summarising the data.	Methods – Data Synthesis

Section	PRISMA-ScR Item	Checklist Item	Reported on Page/Section
Results	14	Give numbers of sources screened, assessed, and included, with reasons for exclusions, ideally using a flow diagram.	Results – Literature Search and Study Selection; Figure 1
Results	15	Present characteristics of included sources of evidence.	Results; Table 1
Results	16	If done, present critical appraisal data of included sources.	Not applicable
Results	17	Present relevant data charted from included sources of evidence.	Results; Table 1
Results	18	Summarise and/or present the charting results in relation to the review objectives.	Results
Discussion	19	Summarise the main results including overview of concepts, themes, and evidence types.	Discussion
Discussion	20	Discuss limitations of the scoping review process.	Study Limitations
Discussion	21	Provide a general interpretation of the results and implications.	Conclusion
Funding	22	Describe sources of funding for included sources of evidence and the scoping review.	Funding
